# Self Help Plus: study protocol for a cluster-randomised controlled trial of guided self-help with South Sudanese refugee women in Uganda

**DOI:** 10.1017/gmh.2018.17

**Published:** 2018-08-13

**Authors:** F. L. Brown, K. Carswell, J. Augustinavicius, A. Adaku, M. R. Leku, R. G. White, P. Ventevogel, C. S. Kogan, C. García-Moreno, R. A. Bryant, R. J. Musci, M. van Ommeren, W. A. Tol

**Affiliations:** 1 War Child Holland, Amsterdam, The Netherlands; 2 Department of Global Health and Population, Research Program for Children and Global Adversity, Harvard T. H. Chan School of Public Health, Boston, MA, USA; 3 Department of Mental Health and Substance Abuse, World Health Organization, Geneva, Switzerland; 4 Department of Mental Health, Johns Hopkins Bloomberg School of Public Health, Baltimore, MD, USA; 5 HealthRight International, New York, NY; 6 Arua Regional Referral Hospital, Arua, Uganda; 7 Institute of Psychology Health and Society, University of Liverpool, Liverpool, UK; 8 Public Health Section, United Nations High Commissioner for Refugees, Geneva, Switzerland; 9 School of Psychology, University of Ottawa, Ottawa, Canada; 10 Department of Reproductive Health & Research, World Health Organization, Geneva, Switzerland; 11 University of New South Wales, Sydney, Australia

**Keywords:** Armed conflict, humanitarian emergencies, interventions, mental health, psychological intervention, trial protocol

## Abstract

**Background.:**

Exposure to armed conflict and forced displacement constitute significant risks for mental health. Existing evidence-based psychological interventions have limitations for scaling-up in low-resource humanitarian settings. The WHO has developed a guided self-help intervention, Self Help Plus (SH+), which is brief, implemented by non-specialists, and designed to be delivered to people with and without specific mental disorders. This paper outlines the study protocol for an evaluation of the SH+ intervention in northern Uganda, with South Sudanese refugee women.

**Methods.:**

A two-arm, single-blind cluster-randomised controlled trial will be conducted in 14 villages in Rhino Camp refugee settlement, with at least 588 women experiencing psychological distress. Villages will be randomly assigned to receive either SH+ with enhanced usual care (EUC), or EUC alone. SH+ is a five-session guided self-help intervention delivered in workshops with audio-recorded materials and accompanying pictorial guide. The primary outcome is reduction in overall psychological distress over time, with 3 months post-treatment as the primary end-point. Secondary outcomes are self-defined psychosocial concerns, depression and post-traumatic stress disorder symptoms, hazardous alcohol use, feelings of anger, interethnic relations, psychological flexibility, functional impairment and subjective wellbeing. Psychological flexibility is a hypothesised mediator, and past trauma history and intervention attendance will be explored as potential moderators.

**Discussion.:**

This trial will provide important information on the effectiveness of a scalable, guided self-help intervention for improving psychological health and wellbeing among people affected by adversity.

**Trial Registration:**

ISRCTN50148022; registered 13/03/2017.

The world is experiencing unprecedented rates of forced displacement due to armed conflicts and other humanitarian crises, with a current estimate of 65.6 million displaced people globally (United Nations High Commissioner for Refugees, 2017). Exposure to armed conflict, displacement and other adversities may have detrimental effects on the mental health of affected populations, and lead to increased risk for symptoms of depression (>17%) and post-traumatic stress disorder (PTSD; >15%) (Steel *et al*. 2009). High rates of psychological distress are associated with significant functional impairment, impacts on physical health and reduced ability to care for and adequately protect oneself and dependents. This has significant subsequent effects on communities and health-care resource utilisation (Norris *et al*. 2002; Prince *et al*. 2007). Ongoing stressors such as poverty and gender-based violence (GBV) commonly experienced in humanitarian settings likely also interact with trauma histories as determinants of mental health in displaced populations (Miller & Rasmussen, 2010; Miller & Jordans, 2016). As such, addressing mental health and psychosocial wellbeing is increasingly seen as a priority in humanitarian settings (Inter-Agency Standing Committee, 2007; United Nations High Commissioner for Refugees, 2013; Ventevogel *et al*. 2015).

Evidence exists for the efficacy of psychological treatments such as cognitive–behavioural therapy in treating psychological distress and disorders (Dua *et al*. 2011; Tol *et al*. 2013), and there is an increasing interest in the research on the applicability, acceptability, effectiveness, implementation and dissemination of these interventions across cultures and contexts (Murray *et al*. 2014; Kane *et al*. 2016). Yet to date, the vast majority of research on mental health interventions for populations exposed to adversity has been conducted in high-income settings (Saxena *et al*. 2007). Significant gaps exist in access to mental health services in low- and middle-income countries (LMICs) and most people in low-resource settings with mental health problems, including refugees, currently do not receive evidence-based care (Saxena *et al*. 2007; Kane *et al*. 2014). In addition, most armed conflicts occur in LMICs (Kim & Conceição, 2010), and these countries also host around 90% of the world's refugees (OECD, 2016). Thus, the damaging effects of armed conflict and displacement frequently lead to increased risk factors and greater mental health needs in the very contexts where health and support systems are greatly challenged to cope with this burden.

For psychological interventions to have promising potential for large-scale implementation in low-resource settings, they must be brief, inexpensive and relatively easy to deliver. Given the dearth of mental health specialists in most regions, particularly in humanitarian crises, scalability can be improved by: (i) further innovating on task-shifting/task-sharing approaches whereby non-specialists are trained and supervised to deliver programmes (Blanchet *et al*. 2013); (ii) enhancing reach *via* approaches targeting a broader array of mental health difficulties simultaneously (Betancourt *et al*. 2014; Murray, 2014; White & Ebert, 2014); and (iii) designing interventions to be more easily adaptable to culture and context (Castro *et al*. 2004; Bernal & Sáez-Santiago, 2006; Castro *et al*. 2010).

To meet these demands, the WHO has published guidelines and evidence-based interventions for use in non-specialised health settings (World Health Organisation, 2015*b*; World Health Organisation, 2016).

## Self Help Plus

In line with the recommendations for stress management interventions (Tol *et al*. 2013) and the need for innovative approaches to address the issues of access and scale, the WHO developed the Self Help Plus (SH+) intervention (Epping-Jordan *et al*. 2016). The programme was developed with experts in psychological intervention and global mental health, with peer review from 43 external experts. SH+ is brief (five sessions) and does not require diagnostic assessment since it aims to target a broad range of psychological difficulties (e.g. depressive and/or anxious mood, stress reactions and client-defined psychosocial problems) that cause distress but do not necessarily meet the diagnostic criteria for a mental disorder. Innovative features include a guided self-help format, comprising an illustrated book and audio materials (which provide the core course content) delivered in a larger group course format, with a guide to assist briefly trained lay facilitators to conduct the course. These materials aim to ensure that key intervention exercises are delivered with fidelity, without the financial and human resource burden of extensive training and supervision. Thus, SH+ may be easier to disseminate and more readily scalable in areas where there is limited access to mental health services.

SH+ is based on Acceptance and Commitment Therapy (ACT), a third-wave cognitive–behavioural approach that incorporates acceptance and mindfulness and encourages meaningful living despite adversity. Specifically, ACT aims to promote psychological flexibility, which is associated with (i) a reduction in attempts to alter or control unwanted internal experiences such as thoughts and emotions (based on the notion that suppressing unwanted thoughts and emotions paradoxically increases them) and (ii) an increased ability to respond adaptively to situations for the purpose of valued living (Hayes *et al*. 2006). Arguments for using ACT in efforts to increase access to mental health support in culturally varied low-resource settings have been highlighted recently (White *et al*. 2017). Several meta-analyses suggest that ACT-based interventions may be effective in various formats, and for numerous psychosocial problems (Hayes *et al*. 2006; A-Tjak *et al*. 2015), including in low-resource and culturally varied settings (Lundgren *et al*. 2006; Stewart *et al*. 2016).

Current progress in psychological research and practice has targeted increased access through innovative delivery models such as psychoeducational courses (Cuijpers *et al*. 2009), e-mental health (Andrews *et al*. 2010) and bibliotherapy (Cuijpers *et al*. 2010). Recent meta-analyses suggest that (i) guided self-help formats may be just as effective as face-to-face interventions for depression (World Health Organisation, 2015*a*); and (ii) self-help mindfulness-based interventions are potentially efficacious in reducing depression and anxiety (Cavanagh *et al*. 2014). Several ACT interventions have been tested in self-help format (Fledderus *et al*. 2012; Jeffcoat & Hayes, 2012; Trompetter *et al*. 2015).

## Setting

This study is part of a larger programme of research being conducted in Rhino Camp refugee settlement, located in northern Uganda. Despite its name, Rhino Camp is not a camp but a set of villages where South Sudanese refugees are able to self-settle on appointed plots of land and utilise existing government health and education services. Most recent figures indicate that approximately 116250 South Sudanese refugees reside in Rhino Camp (V. Kahi, Health Information System Officer, Public Health Section, UNHCR, Geneva).

High rates of mental health problems have been documented in displaced South Sudanese populations, with co-occurring PTSD, depression and anxiety symptoms the most commonly reported (Harsha & Kulkarni, 2014). Local idioms of distress among South Sudanese have also been documented (Ventevogel *et al*. 2013). A recent desk review and needs assessment conducted in Rhino Camp during early phases of this study found high levels of psychological distress among displaced South Sudanese populations. Experiences of GBV, including sexual violence, and early marriage were common. Limited mental health and psychosocial support services were identified. Prominent psychosocial issues identified included psychological distress in the form of ‘overthinking’ and ethnic tensions (Adaku *et al*. 2016).

The study will be conducted with the implementing partner, Peter C. Alderman Foundation (PCAF). PCAF is a non-governmental organisation that has collaborated with the Ministry of Health in Uganda to provide mental health support to conflict-affected populations since 2006 (Nakimuli-Mpungu *et al*. 2013). PCAF has a static clinic at the Arua Regional Referral Hospital, a multi-disciplinary team that visits health centres in Rhino Camp on a weekly basis (psychiatric clinical officer, nurse, counsellor, social worker) and a social worker based in the settlement. At the time of this study, all mental health services in the settlement are provided by PCAF and supervised by a psychiatrist (AA) based in Arua.

## Current study

Our research strategy is informed by the UK Medical Research Council Framework for the Development of Complex Interventions (Craig *et al*. 2008), which recommends an iterative process of: (a) intervention development, (b) feasibility testing and piloting, (c) evaluation and (d) implementation. This framework for the development of interventions emphasises the importance of exploratory and randomised pilot studies prior to large-scale trials, to address uncertainties such as problems of acceptability, compliance, feasibility, delivery of the intervention, recruitment and retention.

In line with this framework, we conducted two preliminary studies: (1) an uncontrolled pilot of SH+ with one group of men and one group of women (Tol *et al*. 2018*a*); and (2) a feasibility cluster-randomised controlled trial (cRCT) with one group of women in the intervention condition, and one group of women in the control condition (Tol *et al*. 2018*b*). Given the concerns of contamination in small communities and with the provision of an illustrated book, a cluster design was chosen. The initial uncontrolled pilot found good adherence among women, promising changes on outcome measures, and encouraging statements of improvement in qualitative interviews. However, adherence among men was suboptimal and a few sessions were disrupted due to some participants attending while intoxicated. We therefore decided that further adaptation was required for use of SH+ with men, and to continue our evaluation of SH+ with women only. Additional details on the translation, adaptation and initial uncontrolled piloting can be found in this volume (Tol *et al*. 2018*a*).

## Methods

### Design

**Fig. 1.**
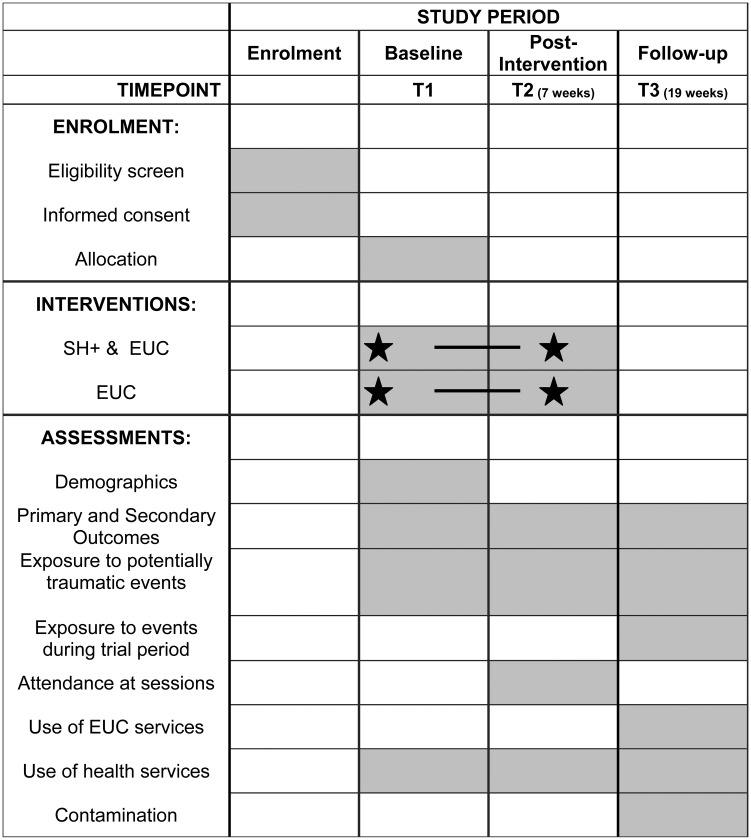
Standard Protocol Items Recommendations for Interventional Trials (SPIRIT): schedule of enrolment, interventions and assessments for cRCT of SH+.

This study is a two-arm, single-blind, superiority cRCT, to evaluate the effectiveness of the locally adapted SH+ alongside enhanced usual care (EUC) (SH+), compared with EUC alone. It is conducted in a community-based setting with South Sudanese refugee women living in northern Uganda. All villages in the zones of Rhino Camp where preliminary studies of SH+ have not been implemented (*n* = 14) will be included and randomisation will occur at the village level such that half of the villages will be allocated to receive SH+ and EUC and half will receive EUC alone. Outcomes on a range of mental health indicators will be assessed at the individual level at baseline (T1), post-intervention (T2; 6 weeks) and 3-month follow-up (T3; 19 weeks). The Standard Protocol Items: Recommendations for Interventional Trials (SPIRIT) is outlined in Fig. 1, and the checklist is attached as an online Supplementary file.

### Aims and hypotheses

The primary aim of this cRCT is to assess the effectiveness of SH+ on symptoms of psychosocial distress at 3 months. The secondary aim is to assess SH+ effectiveness using other measures of mental health and wellbeing from pre- to post-intervention, and at a 3-month follow-up. Additional aims are to assess: (1) whether psychological flexibility acts as a mediator of changes on other outcomes; (2) whether treatment effects are moderated by past experience of sexual and other forms of GBV, the number of different types of potentially traumatic events experienced and attendance at sessions. Health service use will be measured as an index of costs to enable preliminary cost-effectiveness analysis. We will assess fidelity to the intervention manual and contamination of the control group by exposure to SH+ materials or content.

We expect that women in the SH+ arm will show significantly greater improvements on all outcome measures both at immediate follow-up and 3-month follow-up compared with the EUC arm. In addition, we hypothesise that psychological flexibility will act as a mediator such that the intervention will lead to improvements in psychological flexibility, which in turn are associated with improvements on outcome measures.

Although the study is not powered to conclusively determine the moderation effects, we will conduct exploratory analyses of potential moderators. We expect smaller but still significant treatment effects for women exposed to GBV and higher levels of exposure to other potentially traumatic events. We also expect that treatment effects will be moderated by attendance such that greater attendance is related to larger effects.

### Sample size

Recent meta-analyses of self-help acceptance and mindfulness-based therapies (Cavanagh *et al*. 2014) and psychoeducational depression courses (Cuijpers *et al*. 2009) have found small-to-medium effect sizes for depression symptoms. Although our primary outcome measure is psychological distress, this literature was used as the best estimate of expected effect size. Utilizing the PowerUp! Tool (Dong & Maynard, 2013), a minimum detectable effect size was calculated using an average cluster size of 42 individuals, 14 clusters, intracluster correlation of 0.012, 20% attrition, 80% power, an *α* level of 0.05 and a two-tailed test. With these specifications, the minimum detectable effect size is 0.219 with a total *N* of 588 (294 per condition). In the completed small feasibility cRCT in this population, <20% attrition was observed (Tol *et al*. 2018*a*).

### Participants, screening and randomisation

Participants will include any female adult refugee (aged over 18 years) from South Sudan living within study villages in Rhino Camp who: (1) is experiencing psychological distress based on attaining a score of 5 or more on the Kessler 6 (K6; Kessler *et al*. 2010); (2) can understand spoken Juba Arabic (according to self-report). Exclusion criteria will be determined through a structured screening questionnaire administered by trained research assistants, and will include: (1) imminent risk of suicide or other life-threatening risk; (2) observable signs of a severe mental disorder (e.g. psychosis); (3) inability to understand the basic intervention materials (with items 2 and 3 assessed using an observation checklist).

Within each village, households will be randomly selected by spinning a bottle to decide which direction to start in, approaching the first household in that direction and then approaching every fifth house after that. Within households, we will inquire whether there are Juba Arabic-speaking adult women. If more than one woman meets these requirements, we will randomly select one by drawing numbered slips of paper, and screen the woman who drew the slip numbered as one. Potential participants will be screened for eligibility, and recruitment and screening will continue until two SH+ groups (20–25 people per group, or around 40–50 participants in total) have been identified. Assuming that 60% of participants screened will be eligible and willing to participate in the study [conservatively estimated on eligibility rates of 76% in the uncontrolled pilot (Tol *et al*. under 2018*a*)], we estimate needing to screen approximately 1050 individuals. However, screening will be continued until the target sample is achieved. Based on the population statistics (V.Kahi, Health Information System Officer, Public Health Section, UNHCR, Geneva) and experiences in preliminary studies (Tol *et al*. 2018*a*, *b*), we estimate that recruiting sufficient participants from each of 14 clusters will be feasible. To ensure participant retention in the study, we aim to keep detailed address information and discuss current location with family members if participants have moved.

After baseline, simple randomisation of villages will be conducted via software by staff at Johns Hopkins University not involved in the study, and they will reveal allocations to the local implementation team who will inform refugee leaders and individual participants which condition their village has been allocated to, in preparation for intervention commencement. Allocation of villages will not be revealed to the independent assessment team until the end of the trial.

### Outcome measures

The primary outcome is psychological distress across time (T1, T2, T3). Secondary outcomes are: self-defined psychosocial concerns, symptoms of depression and PTSD, hazardous alcohol use, feelings of anger, interethnic group relations, psychological flexibility, functional impairment and subjective wellbeing. The primary end-point is the 3 month follow-up (T3). However, we will also examine the effects of the intervention between baseline (T1) and post-treatment (T2). All measures have been systematically translated from English to Juba Arabic according to standard procedures (van Ommeren *et al*. 1999) and piloted. Psychometric properties were found to be suitable in the preliminary studies, and internal consistencies (using Cronbach's *α*s) are reported below in parentheses. Socio-demographic data will be collected through questions A1–A5 of the WHO Disability Assessment Schedule 2.0 (WHODAS; World Health Organisation, 2010). Outcomes will be assessed through one-to-one interviews in participant homes. These will be conducted by an assessment team, comprised of trained research assistants with strong Juba Arabic and English language skills, and an independent assessment team leader. To accommodate low literacy, pictorial flashcards will be used to depict answering options for the outcome measures. These have been used in preliminary studies and are well understood.

#### Primary outcome: psychological distress

We will measure psychological distress using the K6 (Kessler *et al*. 2010) (*α* = 0.64). This is a brief six-item scale of non-specific psychological distress, screening for the presence of serious mental illness. It has been used in the WHO World Mental Health Surveys and validated in many different countries. Scores range from 0 to 24, and in most applications, a score of 13 or above has been interpreted as indicating a probable serious mental illness (Kessler *et al*. 2003), whereas a score of 5 or more is indicative of moderate or severe psychological distress (Prochaska *et al*. 2012). We will use the K6 as both a screener and an outcome measure.

#### Secondary outcomes

We will assess self-defined psychosocial goals using the Psychological Outcome Profiles instrument (PSYCHLOPS; Robinson *et al*. 2004) (*α* = 0.82). This consists of four questions and three domains: problems (two questions), function (one question) and wellbeing (one question). Participants are asked to give free-text responses to the problem and function domains. Responses are scored on a six-point scale producing a maximum score of 18. The pre- and post-therapy versions of PSYCHLOPS consist of the same four questions but the post-therapy version adds an overall evaluation question (determining self-rated outcome ranging from ‘much better’ to ‘much worse’). PSYCHLOPS has been validated in primary care populations across several countries (Czachowski *et al*. 2011; Héðinsson *et al*. 2012).

We will administer the abbreviated six-item version of the PTSD Checklist-Civilian (PCL-C; Lang & Stein, 2005) (*α* = 0.64) to assess PTSD symptoms. The PCL-C scale uses a five-point response scale, to give a total score ranging between 6 and 30 with higher scores indicating higher levels of PTSD symptoms. It has been well validated across cultures.

To assess depression symptoms, we will use the Primary Health Questionnaire nine-item version (PHQ-9; Kroenke *et al*. 2001) (*σ* = 0.85). The PHQ-9 scale uses a four-point response scale, giving a total score between 0 and 27, with higher scores indicating more depression symptoms. The PHQ has been previously used with South Sudanese internally displaced people (Kim *et al*. 2007).

We will assess hazardous alcohol use through two survey questions designed for the purpose of this study, asking how many days in the last week the participant drank alcohol and how many days they became intoxicated. We will use the addition of the number of days for both questions as a continuous variable.

To assess anger, we will use a shortened version of the explosive anger index, which was developed by Silove *et al.* for use in post-conflict Timor-Leste (Silove *et al*. 2017) and with perinatal women (Silove *et al*. 2015). Our shortened version asks two questions to identify whether participants have experienced attacks of explosive anger (presence score). Participants who endorse these items, will be asked further questions about frequency, what triggers attacks and whether attacks are associated with verbal or physical violence (severity score).

To assess ethnic relations, we developed three questions (*α* = 0.87) that ask about frequency of interacting with people from other ethnicities, in terms of greeting and having conversations in public places, and meeting in one's home. Questions have a four-point response format ranging from 0 (never) to 3 (very often). We will sum answers to form a continuous variable ranging between 0 and 9.

We will assess functional impairment using the WHODAS 2.0, a 12-item interview-administered version (World Health Organisation, 2010) (*α* = 0.82). This instrument assesses health and disability across all health conditions, is applicable across cultures, can be used in all adult populations and has been used in Uganda (Nyirenda *et al*. 2013). WHODAS 2.0 covers six domains (cognition, mobility, self-care, getting along, life activities and participation). It assesses difficulties people have across these domains during the last 30 days.

To assess subjective wellbeing, we will administer the WHO-5 Wellbeing Index, a five-item questionnaire measuring current psychological wellbeing and quality of life (Bech *et al*. 2003) (*α* = 0.80). Scores range from 0 to 25. The scale has demonstrated sensitivity to change in wellbeing and is available in numerous languages (Bech *et al*. 2003).

To assess psychological flexibility, we will deliver the Acceptance and Action Questionnaire (AAQ-II; Bond *et al*. 2011) (*α* = 0.82), a seven-item scale, using a seven-point response scale. Scores range from 0 to 49, with higher scores indicating higher psychological flexibility. It has been used in post-conflict settings (Kashdan *et al*. 2009). Psychological flexibility will be included both as a secondary outcome and as a mediator of the primary and other secondary outcomes.

#### Moderators

To assess the level of exposure to different potentially traumatic events, we will administer an adapted 23-item version of the Harvard Trauma Questionnaire Part A (HTQ; Mollica *et al*. 1992) (*α* = 0.71). Respondents are asked whether they have experienced each of the events. For this study, several items were removed and others added based on contextual relevance in consultation with the local research and clinical team, and one item on torture was adapted from the original version of the HTQ. Two items on the HTQ Part A assessing domestic violence and sexual assault were replaced by three adapted items from the WHO Violence Against Women measure (World Health Organisation, 2005) that were perceived to enhance the ability of the scale to capture these experiences.

At T3, a single question will be asked about any potentially stressful or upsetting events participants have experienced during the trial period. Responses will be coded with general categories (e.g. violence, riots, hunger, destruction of home or property). PCAF reports will be used to identify additional community-level events that may affect particular villages.

A measure of attendance at sessions will be collected via session attendance sheets kept by intervention facilitators.

#### Use of services

To assess the use of EUC services by participants in both trial arms, an identifier will be added to the PCAF routine assessment to indicate whether the participant is in the SH+ trial. At the conclusion of the trial period, data will be gathered on access to any PCAF service (i.e. assessments, group support psychotherapy, medication, social work home-visits, counselling or group health talks).

To assess other health service usage, participants will be asked to list any health service they used for any health problem in the past month, including traditional healers. They will then be asked to identify expenditures in the past month on healthcare, through a series of nine questions.

### Enhanced usual care

EUC will be provided to participants in both SH+ villages as well as participants in control villages. We selected EUC as the comparator to avoid possible nocebo effects associated a waitlist condition (Furukawa *et al*. 2014), while providing more substantial support than usual care. It will consist of an individual visit from a Community Psychosocial Assistant (CPA; a trained Village Health Team member who is a South Sudanese refugee), employed on a small facilitation fee. The CPA will be aware of the allocation of the village and will provide information to all participants over one session of approximately 10–15 min held in the participant's home and covering: the effects of psychological distress; simple strategies to manage ‘overthinking’ (such as physical exercise, regular sleep and keeping a regular routine); services available via PCAF and how to access them. The CPAs will be of mixed sex. Other services will not be restricted in any way to participants in either condition, but will be monitored.

The standard PCAF services include assessments, and then based on need and preferences: psycho-education, group and individual psychological interventions, social work home visits, counselling, medication and group health talks. For SH+ participants, the CPA will also provide details of the SH+ programme and schedule of sessions.

### SH+ implementation

The intervention will involve participants attending five weekly workshop sessions (20–25 people) lasting approximately 2 h each, during which pre-recorded audio materials adapted for the local context will be presented, with participants engaging in several experiential exercises and small group discussions. Participants will also be provided with a locally adapted illustrated self-help book to be used outside the sessions. Two facilitators will conduct the workshop, but their involvement will be minimal. Primarily, their role will be to coordinate the group process, for example, stopping and starting the audio, reading discussion exercises and answering basic questions from participants. The content of the intervention is delivered via the pre-recorded materials, with facilitators trained not to provide detailed explanations, in order to ensure fidelity and keep the need for their training and supervision minimal. A written facilitator guide helps facilitators to conduct the course.

**Fig. 2.**
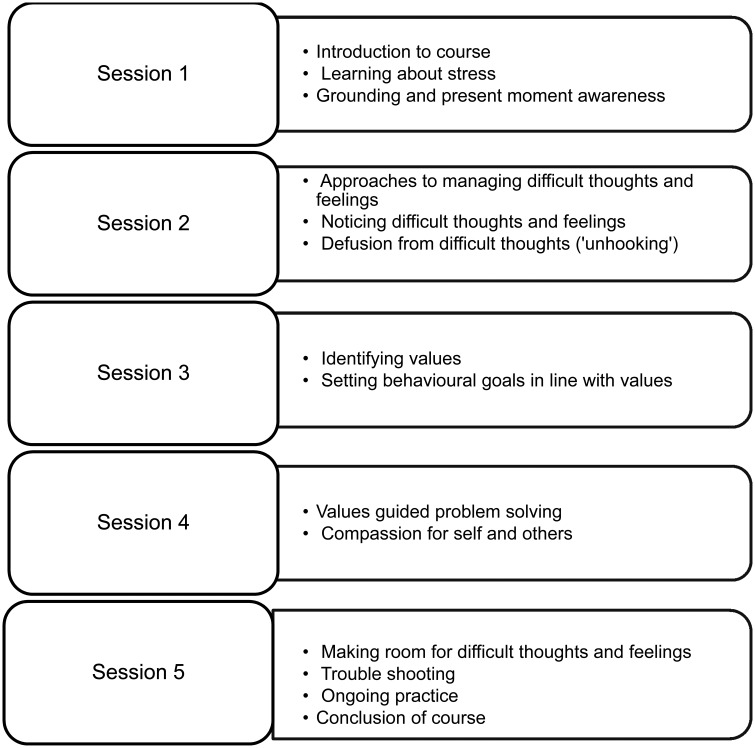
Outline of SH+ programme.

SH+ involves teaching participants skills of: present moment awareness and grounding, defusion from and acceptance of difficult thoughts and feelings, identifying valued life directions and taking action in line with those, and compassion for self and others. A brief outline of the five sessions of SH+ is provided in Fig. 2. Skills learned in any session are reinforced in subsequent weeks.

In Rhino Camp, the audio material will be presented in Juba Arabic – the most common language spoken among South Sudanese. The SH+ book is largely pictorial because of high rates of illiteracy among South Sudanese refugees, but still contains some text. Pilot testing revealed that literate family members may read the book to illiterate course participants between sessions (Tol *et al*. 2018*a*). The book will be offered to participants in either English (a language increasingly understood by young people) or Juba Arabic. Incentives will not be provided for participants to attend SH+ sessions; however, a soda or water, and a biscuit, will be provided to each participant during each SH+ session due to the length of the sessions. Sessions will be held in tent structures erected specifically for this programme, and mobilisation activities will occur prior to each session.

### Facilitator selection, training and supervision

SH+ facilitators have a minimum of completed secondary education, prior experience with psychosocial activities or community mobilisation and reasonable proficiency in both Juba Arabic (spoken) and English language (written and spoken). Four female facilitators from Arua (Uganda) were employed for the duration of the initial uncontrolled pilot study and prior feasibility cRCT. Training for these facilitators comprised a 5-day training prior to the uncontrolled pilot study and a further 4 days of training prior to the feasibility cRCT because of substantial changes to the SH+ package based on the results of the pilot. This training was conducted by a WHO master trainer (KC). The training provided information on psychological distress, taught skills in identifying and managing participant distress and managing group processes, explained the aims and background to the SH+ intervention, and allowed facilitators to experience taking part in the course themselves. PCAF clinical team members also attended this training, to prepare them to supervise the overall conduct of the intervention and contribute to general capacity building. PCAF clinical team members do not use SH+ techniques, audio-recordings or books in routine services, and the general concepts of ACT and SH+ were not covered sufficiently in training to enable them to be used without materials; therefore, contamination of EUC was not considered an issue.

Competency checks were completed during the training and prior to the feasibility cRCT. These comprised of facilitators completing two role-plays each (one of running an SH+ group session and the other supporting a distressed participant), chosen by the WHO master trainer.

After the feasibility cRCT but prior to the current cRCT, a further four female facilitators from the same area will be employed. The training for these facilitators will be provided in two stages of 4 days each. The first stage will be conducted by the previously trained team who gained experience with SH+ during preliminary studies. This stage will mainly involve listening through the audio course and reading the accompanying book, along with initial practice in running groups. The second stage of the training will be provided by the facilitator team leader in conjunction with the WHO master trainer and focus on the skills covered in the pilot training described above. This will be followed by the same competency assessment. This two-stage approach will also build training capacity in the local facilitator team.

A social worker from PCAF will supervise the conduct of SH+ during the cRCT. The clinical supervisor and the facilitator team leader will receive remote support and supervision on an as needed basis (but no more than 1 h per week) from the WHO master trainer.

**Fig. 3.**
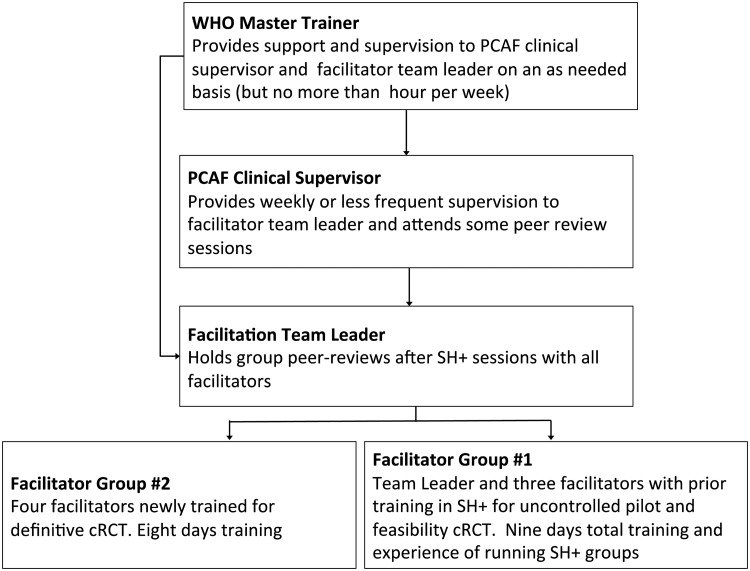
Structure of the SH+ intervention delivery, supervision and training team.

Protocol adherence will be ensured through group peer-review sessions after each SH+ session. Peer reviews will cover potential difficulties encountered in delivering SH+, feedback on participant or facilitator concerns, and any adverse events (AEs; e.g. injuries on the way to treatment, increase in distress) and serious adverse events (SAEs; e.g. suicide attempts, serious violence). The facilitator team leader will receive supervision from the clinical supervisor weekly or less frequently, with the supervisor also attending some peer-review sessions to provide support. The structure of the SH+ intervention delivery, supervision and training team is illustrated in Fig. 3.

### SH+ fidelity

Fidelity will be assessed using adherence monitoring checklists to note any deviations from protocol (i.e. a checklist of all activities to be completed in each workshop according to the intervention manual) by both facilitators present at each workshop. Any deviations will be reported to the WHO master trainer after supervision. The clinical supervisor will directly observe a sample of at least 10% of all SH+ workshops, and will complete the same fidelity checklist.

### Ethics and trial procedures

Ethical approval has been obtained from the WHO Ethics Review Committee (ERC), the MildMay Uganda Research Ethics Committee and the Uganda Council for Science and Technology. Permission to conduct research and mental health support activities has been provided by the Office of the Prime Minister in Uganda and the United Nations High Commissioner for Refugees in Uganda.

Potentially eligible women identified through the recruitment process described above will be given oral and written information in the local language about participating in the screening process by a research assistant, who will then seek informed consent for screening. For eligible participants, oral and written information about the trial will be provided by research assistants, and participants will be asked to complete a written consent form. For participants who are illiterate, witnessed oral consent and a thumb print will be considered sufficient. Participants will be free to decline to participate or withdraw without any effect on their routine care.

Small non-financial incentives (e.g. a package of soap) will be provided to compensate participants’ for their time in completing outcome assessments. In case participants do not attend a scheduled assessment, three attempts will be made to contact them to schedule a new appointment, via home visits or contacting other members of the community.

All AEs and SAEs will be recorded by the research team and reported to a data safety monitoring board (DSMB) consisting of an external clinical officer, an external social worker, the project coordinator and the independent assessment team leader. This will occur within 24 h for SAEs and as soon as possible for AEs. A representative from the DSMB will review SAEs within 48 h and, in addition, the DSMB will review all AEs at least twice a month. If necessary, appropriate action will be taken with respect to individual participants, or conduct of the trial (such as referral to specialised care, installing extra assessment points for monitoring participants or discontinuation). No interim analyses are planned. The local project coordinator is responsible for ensuring timely follow-up of any SAEs, and will inform the participants and DSMB if any data indicate that the disadvantages of participation may be significantly greater than expected.

### Blinding and contamination

Participants and implementation staff will not be blind to village allocation. The independent assessment team will remain blind to the intervention allocation of villages throughout the trial, and will operate independently from the intervention team (with offices in separate parts of Arua). All staff have been trained and supervised in the importance of maintaining blinding, and at no time will intentional unblinding of the independent assessment team be required. Prior to conducting each post-intervention and follow-up assessment, instructions will be given by assessors to all participants about the importance of not revealing their village allocation.

Contamination assessments with 15% of participants in each cluster will be conducted at the 3-month follow-up. As these assessments will reveal village allocation, they will be conducted by SH+ facilitators rather than research assistants. Data entry assistants will enter the data into computer systems, and this contamination assessment data will only be entered once all outcome assessments have been completed to prevent unblinding.

Should blinding be compromised for a particular participant, the independent assessment team leader will be alerted. If this occurs during an assessment, the assessment will immediately be halted and a new research assistant will conduct the rest of the assessment. Such assessments will be marked as being conducted by a different research assistant for analysis purposes.

### Statistics

All analyses will be detailed in a statistical analysis plan, which will be signed before unmasking the study data set. As a first step, we will assess the comparability of study conditions at baseline (demographic characteristics, scores on moderators and mediators at baseline) using χ^2^ with continuity correction or Fisher's exact test for frequencies, and independent-sample *t* tests for continuous measures. In the case of any imbalance, we will correct using propensity scoring. We will explore the distributional properties of the outcome variables at all time points and adjust if needed (e.g. using log transformation). Also as a preliminary step, we will analyse crude mean changes on the outcome measures between groups, not corrected for clustering at the village level. This will involve calculating change scores between (T1−T2, T2−T3, T1−T3) scores for the SH+ and EUC groups separately on an intent-to-treat basis (last observation carried forward). These crude change scores will be compared using independent-sample *t* tests, and considered exploratory analyses only.

To test our hypotheses, we will use latent growth curve modelling (LGCM) in a structural equation modelling framework (Duncan & Duncan, 2004). LGCM will be applied to examine statistically significant differences in longitudinal trajectories on outcome measures between the SH+ and EUC groups (over the three time points: T1, T2 and T3). LGCM allows for the modelling of growth processes using participant-specific random intercepts and slopes. The benefit of this approach is that it accounts for clustering as recommended by the CONSORT statement for cRCTs (Campbell *et al*. 2004), builds on data at all time points simultaneously and allows for sophisticated missing data handling.

LGCM will be conducted in three steps. First, we will model growth curves, using all time points [T1 (0 weeks), T2 (7 weeks) and T3 (19 weeks)], and estimate the intervention effect of SH+, compared with EUC alone, on changes over time on the following outcomes: psychological distress (primary outcome), functional impairment, hazardous alcohol use, feelings of anger, interethnic group relations, self-defined psychosocial goals, depression symptoms, PTSD symptoms, psychological flexibility and subjective wellbeing.

Second, we will add potential moderators and their interaction effects to explore variations in intervention effects. Trajectories of outcome measures will be compared between study conditions, while taking into account interaction effects with the following potential moderators of treatment effectiveness: exposure to GBV, trauma exposure to a large range of potentially traumatic events and attendance at sessions. As a secondary analysis, we will test whether baseline levels and types of distress act as moderators. This will be accomplished by creating interaction terms between study condition and moderators of interest. Significant interaction effects will be further probed utilizing model test statements.

Third, a mediation analysis will be conducted to determine whether increases in psychological flexibility with SH+, mediate improvements on: distress, functional impairment, hazardous alcohol use, feelings of anger, interethnic group relations, self-defined psychosocial goals, depression symptoms, PTSD symptoms and subjective wellbeing. In order to assess these mediation effects, we will conduct separate parallel process LGCM analyses (Cheong *et al*. 2003). A parallel process LGCM characterises participant-specific growth processes for a mediator and outcome variable simultaneously, and relates the growth processes with each other while also enabling an assessment of the influence of time-invariant and time-varying variables.

We will use full information maximum likelihood estimation (FIML) as implemented in Mplus 8.15 (Muthén & Muthén, 1998–2017) to adjust the estimates of the parameters to reflect missingness. FIML is considered the appropriate method for handing data missing at random (Schafer & Graham, 2002). Data will be checked prior to the implementation of FIML to address the assumption of missing at random. Results will be presented using point estimates, *p* values, odds ratios (when relevant) and 95% confidence intervals. Difference testing will be conducted to determine if the sample completing the intervention and follow-up assessments is significantly different from those who were lost to follow-up, in basic demographics as well as baseline variables.

Contamination within the EUC village participants (i.e. access to SH+ materials, or other content or messages) will be analysed descriptively. If substantial contamination is identified, contamination-adjusted analyses will be conducted.

In terms of cost-effectiveness, we will apply a societal perspective on costs, including cost of services utilised by participants and losses in productivity. Primary analysis will be on total costs in previous 3 months at T1 and T3. Bootstrap sampling will be repeated 1000 times on skewed cost data. Cost-effectiveness ratios will be calculated by combining total costs with the different effectiveness measures.

Data will be double-entered from paper copies, and data management and descriptive analyses will be conducted in STATA 14.1 (StataCorp, 2015). Analyses testing hypotheses will be conducted using MPlus 8.15 (Muthén & Muthén, 1998–2017) and will be reported according to the CONSORT guidelines for cRCTs.

### Trial management

The field-based research team will consist of research assistants, independent assessment team leader, overall project coordinator and an independent trial consultant. The principal investigator (WT) will support the trial by communicating weekly with the trial team. The independent consultant will be experienced with trial management in Uganda and through two field visits, will check and document whether all aspects of the project are correctly implemented (e.g. completing a checklist of whether study implementation adheres to standard operating procedures, including whether all assessments are completed on time, blindness is maintained and collected data are legible and correctly entered and stored). Narrative reports will be provided every 3 months and regular visits to the study site will be conducted by the project management team. We will continue to coordinate activities with the Office of the Prime Minister and UNHCR in Uganda.

## Discussion

As a guided self-help programme, SH+ has been developed with the aim of reducing the global treatment gap for psychological interventions, by providing a scalable solution that has the potential to reach many individuals currently without access to mental health support, with relatively little investment. The delivery format is innovative since fidelity to the core content of the intervention is ensured via pre-recorded locally adapted audio material as well as an illustrated book. Training and supervision requirements are also reduced. Preliminary studies in northern Uganda indicate that SH+ can be feasibly adapted and is considered appropriate and useful by participants (Tol *et al*. 2018*a*). This cRCT will assess the effectiveness of SH+ delivered by non-specialist facilitators for female South Sudanese refugees, living in northern Uganda. An important avenue for future research is further exploration of the necessary adaptations required to increase the suitability of SH+ with male participants. If sufficient evidence is established, the SH+ materials will be published by the WHO and will be made publicly available on its website. Future work should specifically investigate the scalability of this approach and adaptations for specific populations.

## Trial status

Trial recruitment commenced in March 2017 and T3 data collection is in progress. Results of this study are expected in late 2017. Access to the full study protocol and final data set will be available from corresponding author on reasonable request.

## Supplementary material

For supplementary material accompanying this paper visit http://dx.doi.org/10.1017/gmh.2018.17. click here to view supplementary material
